# Engineered IRES-mediated promoter-free insulin-producing cells reverse hyperglycemia

**DOI:** 10.3389/fendo.2024.1439351

**Published:** 2024-08-30

**Authors:** Yumin Li, Doulathunnisa Ahamed Younis, Cong He, Chengming Ni, Rui Liu, Yunting Zhou, Zilin Sun, Hao Lin, Zhongdang Xiao, Bo Sun

**Affiliations:** ^1^ State Key Laboratory of Digital Medical Engineering, School of Biological Science and Medical Engineering, Southeast University, Nanjing, Jiangsu, China; ^2^ Department of Immunology, School of Medicine, UConn Health, Farmington, CT, United States; ^3^ Jiangsu Key Laboratory for Bio functional Molecules, College of Life Science and Chemistry, Jiangsu Second Normal University, Nanjing, China; ^4^ Department of Endocrinology, Zhongda Hospital, Institute of Diabetes, School of Medicine, Southeast University, Nanjing, Jiangsu, China; ^5^ Department of Genetic Engineering, College of Natural Science, University of Suwon, Hwaseong, Kyunggi-Do, Republic of Korea; ^6^ Department of Endocrinology, Nanjing First Hospital, Nanjing Medical University, Nanjing, Jiangsu, China

**Keywords:** IRES, CRISPR/Cas9, promoter-free, insulin-producing cells, diabetes

## Abstract

**Background:**

Endogenous insulin supplementation is essential for individuals with type 1 diabetes (T1D). However, current treatments, including pancreas transplantation, insulin injections, and oral medications, have significant limitations. The development of engineered cells that can secrete endogenous insulin offers a promising new therapeutic strategy for type 1 diabetes (T1D). This approach could potentially circumvent autoimmune responses associated with the transplantation of differentiated β-cells or systemic delivery of viral vectors.

**Methods:**

We utilized CRISPR/Cas9 gene editing coupled with homology-directed repair (HDR) to precisely integrate a promoter-free EMCVIRES-insulin cassette into the 3’ untranslated region (UTR) of the GAPDH gene in human HEK-293T cells. Subsequently quantified insulin expression levels in these engineered cells, the viability and functionality of the engineered cells when seeded on different cell vectors (GelMA and Cytopore I) were also assessed. Finally, we investigated the therapeutic potential of EMCVIRES-based insulin secretion circuits in reversing Hyperglycaemia in T1D mice.

**Result:**

Our results demonstrate that HDR-mediated gene editing successfully integrated the IRES-insulin loop into the genome of HEK-293T cells, a non-endocrine cell line, enabling the expression of human-derived insulin. Furthermore, Cytopore I microcarriers facilitated cell attachment and proliferation during *in vitro* culture and enhanced cell survival post-transplantation. Transplantation of these cell-laden microcarriers into mice led to the development of a stable, fat-encapsulated structure. This structure exhibited the expression of the platelet-endothelial cell adhesion molecule CD31, and no significant immune rejection was observed throughout the experiment. Diabetic mice that received the cell carriers reversed hyperglycemia, and blood glucose fluctuations under simulated feeding stimuli were very similar to those of healthy mice.

**Conclusion:**

In summary, our study demonstrates that Cytopore I microcarriers are biocompatible and promote long-term cell survival *in vivo*. The promoter-free EMCVIRES-insulin loop enables non-endocrine cells to secrete mature insulin, leading to a rapid reduction in glucose levels. We have presented a novel promoter-free genetic engineering strategy for insulin secretion and proposed an efficient cell transplantation method. Our findings suggest the potential to expand the range of cell sources available for the treatment of diabetes, offering new avenues for therapeutic interventions.

## Introduction

Diabetes mellitus (DM) is a complex and heterogeneous disease with an increasing prevalence worldwide. Projections indicate that the number of diabetic patients will reach 693 million by 2045 (1), continuing to rise at an alarming rate and becoming a significant global health burden (2, 3). The dysfunction of islet β-cells is a critical factor in the pathogenesis of diabetes. In type 1 diabetes (T1D), these cells are targeted by auto-reactive T cells, leading to a loss of 70–90% of β-cell mass and a consequent reduction or cessation of insulin secretion (4). In type 2 diabetes (T2D), environmental factors such as malnutrition or obesity impair insulin function, accelerating the depletion of islet β-cells (5). Despite ongoing research and advances in medication and treatment, DM remains a critical health issue due to its associated complications. Prolonged hyperglycemia can lead to severe pathological conditions, including renal failure, cardiovascular diseases, metabolic syndrome, and hormone dysfunctions (6, 7). The limitations of organ transplantation, including donor shortages and the requirement for immunosuppressive drugs (8, 9), make regular insulin administration the primary treatment for diabetes (10).

However, the burden of frequent insulin injections underscores the need for less invasive methods of exogenous insulin delivery or the restoration of β-cell function. These approaches offer great promise for achieving long-term glycemic control in the treatment of diabetes. Insulin-secreting cells generated from stem cells (11, 12) or through genetic engineering of various cell types have emerged as advanced alternative therapeutic strategies for diabetes (13–17). However, it is challenging that not all stem cell lines differentiate with equal efficiency (11). Due to the pluripotent nature of stem cells and the complexity of the differentiation process, there is a risk that unintended or potentially dangerous non-target cell types may persist within the final population of differentiated cells. Of particular concern is the possibility of highly proliferative undefined progenitor cells or residual human pluripotent stem cells, which could pose a tumorigenic risk (18, 19).

Gene-edited engineered cells represent a potentially more convenient and stable alternative to insulin-producing cells that require complex differentiation steps. The implantation of glucose-responsive insulin-expressing elements into extra-pancreatic mammalian cell types could offer protection against DM (20, 21). Previous studies have demonstrated that human embryonic kidney 293T (HEK-293T) cells are capable of producing high levels of anti-diabetic proteins (22–24).

However, current approaches to engineer insulin-secreting cells often rely on viral vectors, where insulin transcription and translation are driven by strong promoters (25, 26). A significant concern with this method is the potential for insertional mutagenesis, which can result from enhancer-mediated dysregulation of adjacent genes or abnormal splicing processes (27). To mitigate these risks, we have designed a promoter-free insulin secretion system using HEK-293T cells.

CRISPR-Cas9 is undoubtedly a powerful gene editing tool for our purposes. This technique allows for precise insertions or deletions within genomic DNA sequences, correcting even genetically mutated cells and tissues. Cells possess several mechanisms for repairing double-strand breaks (DSBs), including non-homologous end-joining (NHEJ), which typically introduces unpredictable mutations, and homology-directed repair (HDR), which involves copying donor DNA strands into DSB regions (28). Genome editing based on HDR is increasingly being studied for its ability to precisely insert DNA fragments, and it has become a well-established and precise gene editing method (29–34).

To achieve promoter-free insulin secretion, we selected the Internal ribosome entry site (IRES) as a key component. This natural translational enhancer, found in various mRNAs, has garnered increased attention due to its ability to initiate cap-independent translation (35–37). The encephalomyocarditis virus (EMCV) IRES, in particular, has been shown to be active in most tissues and organs (37). Consequently, EMCV-IRES-based vectors are frequently employed to co-express multiple therapeutic genes within the same transcription unit, playing a significant role in combined gene therapy (38–43).

In the current study, we successfully integrated a promoter-free IRES-human furin-cleavable human insulin (IRES-hINS) fragment into the GAPDH locus using a CRISPR-Cas9-mediated HDR-based knock-in strategy. This approach resulted in an increase in insulin secretion without altering gene transcription in the cell itself, successfully reversing STZ-induced diabetes in mice over a prolonged period. These findings suggest a highly promising approach in the field of diabetic therapeutics.

## Materials and methods

### Generation of insulin-producing cell lines

#### Cell culture

HEK-293T cell line was purchased from the American Type Culture Collection (ATCC). The cells were cultured in Dulbecco’s modified eagle’s medium (DMEM, D-glucose content 4.5g/L), supplemented with 10% fetal bovine serum (FBS) and 1% of penicillin/streptomycin (100 units/mL penicillin and 100 µg/mL streptomycin) and maintained in a humidified chamber at 37 °C and 5% CO_2_. All cultured medium were obtained from Hyclone Laboratories Inc (Logan, UT, USA).

#### Plasmid construction and generation of EMCVIns

The donor plasmid, ires-eGFP (+HAs) donor-1 (Cat # 87865) with human GAPDH left and right homologous arms was purchased from Addgene. Codon-optimized furin-cleavable human-derived insulin (hIns) was synthesized according to previous reports (44). To be brief, modifications have been made to replace the 62^nd^ arginine to leucine and lysine (29^th^ and 31^st^) to arginine respectively, which could favor the furin-mediated cleavage at B chain junctions of pro-insulin to obtain mature insulin and C-peptide. Then, the green fluorescence protein (GFP) (next to the EMCV-IRES) was replaced with the above-mentioned codon-optimized mCherry-P2A-hInsulin (mchP2AIns) sequences by inserting EcoR I restriction site using the site-directed mutagenesis kit (Vazyme, China) and henceforth called ires-mchP2AIns (+HAs) donor plasmid. Later, the mcherry sequence in the ires-mchP2AIns (+HAs) donor plasmid was replaced with a puromycin DNA sequence to create ires-puroP2AIns (+HAs) donor plasmid. The sgRNA plasmids were constructed by inserting sg1 and sg4 sequences into pCas-Guide-GFP (Origene Cat # GE100012) as per the manufacturer’s instructions and are referred to as Cas-sg1 and Cas-sg4, respectively. The primers used in this study are given in **Supplementary Table 1**.

Cas-sg1 and ires-puroP2AIns (+HAs) donor plasmids were co-transfected into HEK-293T cells using jetPRIME polyplus transfection reagent (Polyplus Transfection, France) following the manufacturer’s protocol. Later, the cells were screened with 10 µg/mL of puromycin for five passages to get pure lines of insulin-producing HEK-293T cells and henceforth named as EMCVIns cells.

### Transfection and integration verification

HEK-293T cells were seeded into 12-well plates at a density of 5 × 10^5^ cells/well and allowed to attach overnight. Then, 1.5 ug DNA (1 ug donor plasmid + 0.5 ug Cas-sgRNA plasmid) and 3 µl jetPRIME polyplus transfection reagent (Polyplus Transfection, France) were used for transfection in each well following the manufacturer’s protocol. After 48h, the successfully integrated cells showed red fluorescence and were imaged using an inverted fluorescence microscope (Nikon, Japan). The efficiency of the successful genomic integration of donor DNA (mchP2AIns) was calculated using Flow cytometer (BD Accuri C6, USA).

The genomic DNA was isolated from both transfected (donor and sgRNA plasmid transfection as mentioned above) and control (without transfection) cells using Multisource Genomic DNA Miniprep Kit (Axygen, USA) following the manufacturer’s instructions. The target site was PCR amplified with different specific primers (**Supplementary Table 2**) and the PCR amplicons were analyzed with Tanaon-4200 Chemiluminescent Imaging System. The successful integration of the donor DNA (mchP2AIns/puroP2AIns) sequences into the precise GAPDH genomic locus was verified using DNA sequencing.

### Immunofluorescence staining

EMCVIns cells were seeded into 12-well plates at a density of 5 × 10^5^ cells/well and allowed to attach overnight. Next, cells were fixed with 4% paraformaldehyde for 15 min, permeabilizated with 0.1% Triton X-100 for 5 min and blocked for 1 h in 3% BSA (Sigma-Aldrich, SRE0096). Subsequently, cells were incubated with an anti-insulin primary antibody (1:100 dilution, Abcam, EPR17359) overnight at 4°C. After 3 times PBST washing, the cells were incubated with Alexa Fluor 488-conjugated secondary antibodies (1:500 dilution, Thermo Fisher Scientific, A32731) for 1 h at 37°C. Next, the cells were washed three times with PBST and counterstained with DAPI (0.5g/ml) for 5 mins at RT. Then the cells were imaged using confocal microscopy (Leica, Germany).

### Insulin secretion assays

#### Cellular Insulin Secretion Assay

HEK-293T cells were seeded into 12–well plates at a density of 5 × 10^5^ cells/well and incubated at 37°C overnight (DMEM, D-glucose content 4.5g/L). About 24h later, the cells were placed with fresh complete medium. 1.5 ug DNA (1 ug mchP2AIns/puroP2AIns donor plasmid+ 0.5 ug Cas-sgRNA plasmid) was transfected using polyplus transfection reagent as mentioned above. About 4 h later, the transfected cells were replaced with fresh complete medium. Then the supernatant was collected after 24h and 48h respectively post-transfection.

#### Glucose-stimulated insulin secretion

Screened pore EMCVIns cells were starved in Krebs-Ringer buffer supplemented with 2mM glucose for 2 h in a 37°C, 5% CO_2_ incubator and were stimulated by 1000μL Krebs-Ringer buffer with low (5mM), middle (11mM) or high (25mM) glucose concentration. Supernatants were collected after 2 h post stimulation. To extract the insulin component from the cells, we treated the cells with 1000 μL of acid-ethanol solution (containing 74% [v/v] ethanol, 1.4% hydrochloric acid, and 24.6% ultrapure water) at 4°C overnight. All the secreted insulin level was measured using a sandwich ELISA kit (ABclonal, Wuhan, China) according to the manufacturer’s instructions. Additionally, the total DNA content of each sample was determined using a DNA Quantification Kit (TIANGEN, China) to standardize insulin secretion.

### Microencapsulation of EMCVIns cells

Cytopore I (GE, USA) and GelMA (Engineering for Life, China) are two common commercial biomaterials to encapsulate cells. Before encapsulation, EMCVIns cells cultured in the 2D system were harvested, labeled with lipophilic tracer DiO (Yeasen, Shanghai, China) for 20 mins at 37°C and washed with D-PBS three times. The Cytopore I biomaterials were soaked in D-PBS, sterilized in high-pressure steam, and followed by washing with D-Hanks and stored in DMEM with 10% FBS before use. An adequate number of primed microcarriers were added to the non-treated tissue culture plate to cover the bottom, to which the DiO-stained EMCVIns cells were then added. Crystal violet staining and CCK-8 (Yeasen, Shanghai, China) kit was used to monitor cell proliferation. At the same time, EMCVIns cells were mixed with GelMA-60 by following the manufacturer’s instructions. GelMA-60 inclusions were labeled with Calcein-AM (Yeasen, Shanghai, China) to identify living cells.

To measure the secreted human insulin in cell culture, the culture supernatants of Cytopore I and GelMA-60 encapsulated cells were collected after 24 h, centrifuged to remove the cell debris, and evaluated by ELISA kit. The empty microcarriers and GelMA-60 were used as controls.

### Mouse studies

8-week-old male C57BL/6 mice were purchased from Beijing Vital River Laboratories and were randomly divided into four groups (n=6 in each group). STZ (Sigma Aldrich, USA) was dissolved in sterile citrate buffer (0.05 M sodium citrate, pH4.5) and injected intraperitoneally into mice (40 mg/kg) for five consecutive days. Control age-matched mice received the same volume of citrate buffer. Fourteen days after the initial STZ injection, serum glucose level was measured every 3 days from tail vein blood using a One-touch glucometer (Roche) in 6 h fasted mice. Mice with serum glucose levels ranging between 12 to 20 mmol/L for continuous 3 days were considered diabetic.

#### Transplantation of EMCVIns cells

About 5 × 10^6^ EMCVIns cells were resuspended in DMEM, taken in a 2 mL syringe, and allowed to sink for a while before being seeded on Cytopore I. The extra medium was then expelled, and the encapsulated microcarriers were subsequently injected into the inguinal fat pad of the mice. Another set of mice received the same number of encapsulated control HEK-293T cells, whereas the control group received empty microcarriers without cells. Serum glucose level was monitored every 3 days after implantation.

#### The intraperitoneal glucose tolerance test

An intraperitoneal glucose tolerance test (IPGTT) was performed on mice on day 14 of post-implantation. Mice were given an intraperitoneal injection of glucose (2 g/kg body weight) after overnight fasting. The glucose levels were measured after the injection at regular intervals of 0, 15, 30, 60, 90, and 120 min, post-glucose injection. Healthy mice served as the control. To measure the human insulin in the mice plasma, mice were anesthetized and the blood was collected from the abdominal aorta followed by a centrifugation at 3000rpm for 10 min to get the serum. The obtained serum was subsequently analyzed with an ELISA kit to determine the quantities of human insulin.

### Statistical analysis

Data were represented as means ± standard deviation (SD). Statistical comparisons were made using Student’s t-test or one-way analysis of variance (ANOVA) and Tukey post-test. Statistical significance was considered if P < 0.05.

## Results

### *In vitro* expression of insulin by mchP2AIns-293 cells

**Figure 1:**
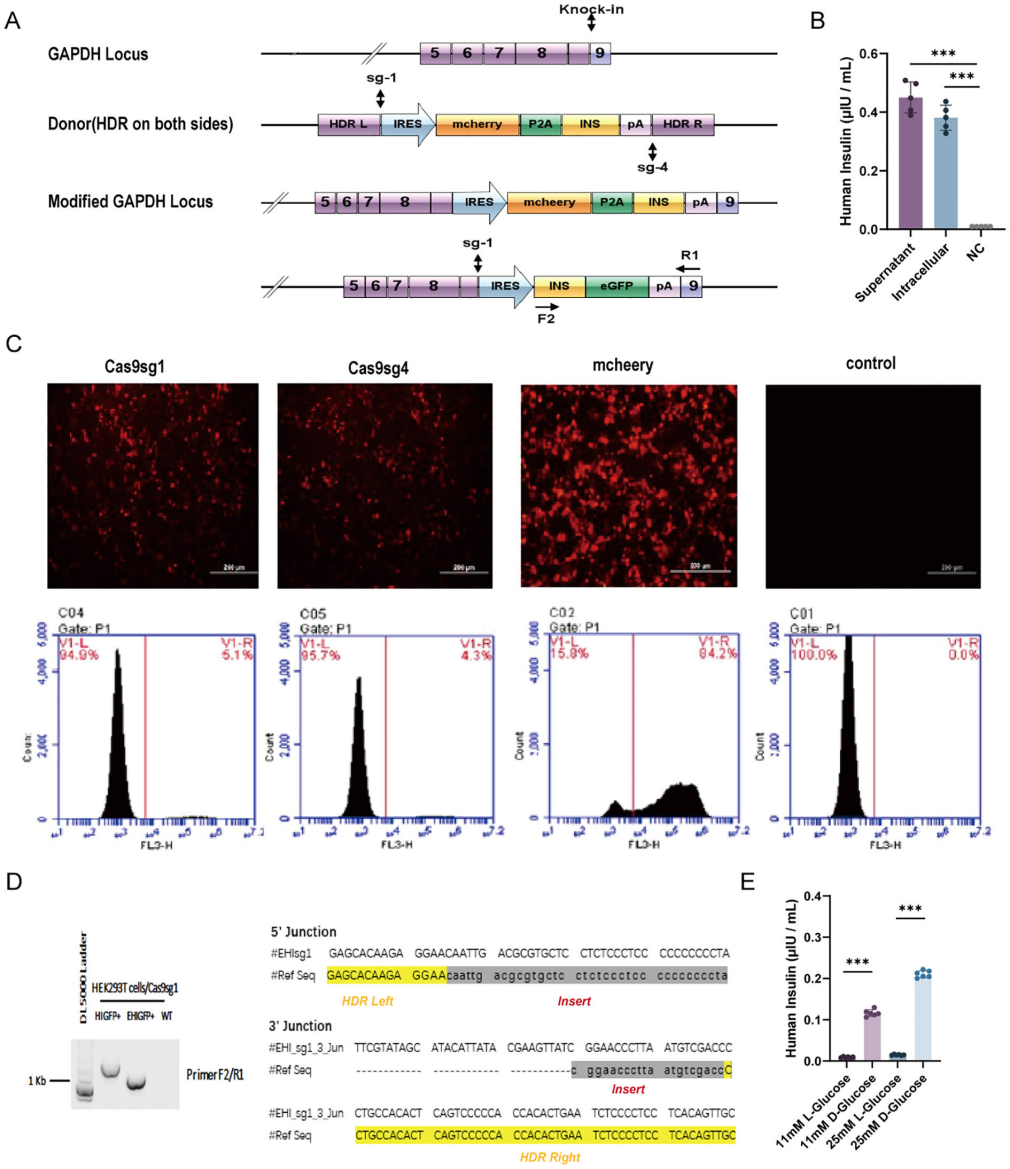
HDR-mediated modified human insulin gene knock-in in HEK293T cells. **(A)** Schematics of the donor plasmid and targeting strategy for HDR-mediated knock-in of the modified human insulin at GAPDH 3′-UTR. Dashed lines indicate sections of homology between the GAPDH genomic locus and donor plasmid DNA. Arrows indicate the positions of PCR primers for insulin integration examination. **(B)** The quantity of insulin in culture media (supernatant) and cell lysis(intracellular) were assessed using ELISA. Fresh culture medium was used as negative control (NC). **(C)** HDR-mediated integration efficiency of Cas9sg1 and Cas9sg4 using fluorescence images and Flow cytometer analysis. Cas9 plasmid without sgRNA was used as a control. **(D)** Genome PCR analysis of mcherry^+^ cells produced with Cas9sg1 in sequencing results of the PCR amplicons with expected modifications (human insulin gene) were integrated precisely at both 5′- and 3′-junctions. **(E)** Insulin secretion from mchP2AIns in response to different types of glucose stimulation. Data are expressed as mean ± SD. n = 5; ***p < 0.001 by student’s t-test.

We successfully engineered insulin-producing cells by integrating a modified insulin gene into the GAPDH locus of HEK-293T cells using CRISPR/Cas9-mediated HDR (**Figure 1A**). A reporter system coupled with the modified insulin gene allowed direct quantification of CRISPR/Cas9-induced HDR-mediated insulin gene integration. We selected two different sgRNA sequences targeting the human GAPDH locus from a previously published report (45). Flow cytometry analysis indicated that integration frequency was slightly higher with Cas9sg1 (4.8–5.1%) compared to Cas9sg4 (4.1–4.3%), with a transfection efficiency of 84.2% (**Figure 1C**). No mCherry-positive cells were detected in the absence of either sgRNA or donor plasmid. Subsequent genomic DNA PCR and sequencing of mCherry-positive cells confirmed the integration of mchP2AIns at the GAPDH 3’UTR, demonstrating HDR-mediated targeting (**Figure 1D**).

To assess whether the integrated modified insulin gene could secrete mature insulin into the culture medium, both mchP2AIns cells and their supernatant were analyzed by ELISA. A fresh culture medium served as a control. The culture supernatant of mchP2AIns cells and the intracellular level showed mature insulin production of 0.45 ± 0.061 μIU·10^5^ cells^-1^·mL^-1^·24 h^-1^ and 0.38 ± 0.06 μIU·10^5^ cells^-1^·mL^-1^·24 h^-1^, respectively, indicating successful synthesis and secretion of mature insulin via CRISPR/Cas9-induced HDR-mediated gene integration (**Figure 1B**).

We then investigated the effect of various glucose concentrations and formulations on EMCV IRES-mediated insulin synthesis. mchP2AIns cells were subjected to glucose stimulation tests with both L- and D-glucose. Notably, the cells responded to D-glucose, which is metabolically active in the human body (**Figure 1E**).

### EMCVIns cells give stable insulin secretion *in vitro*

**Figure 2:**
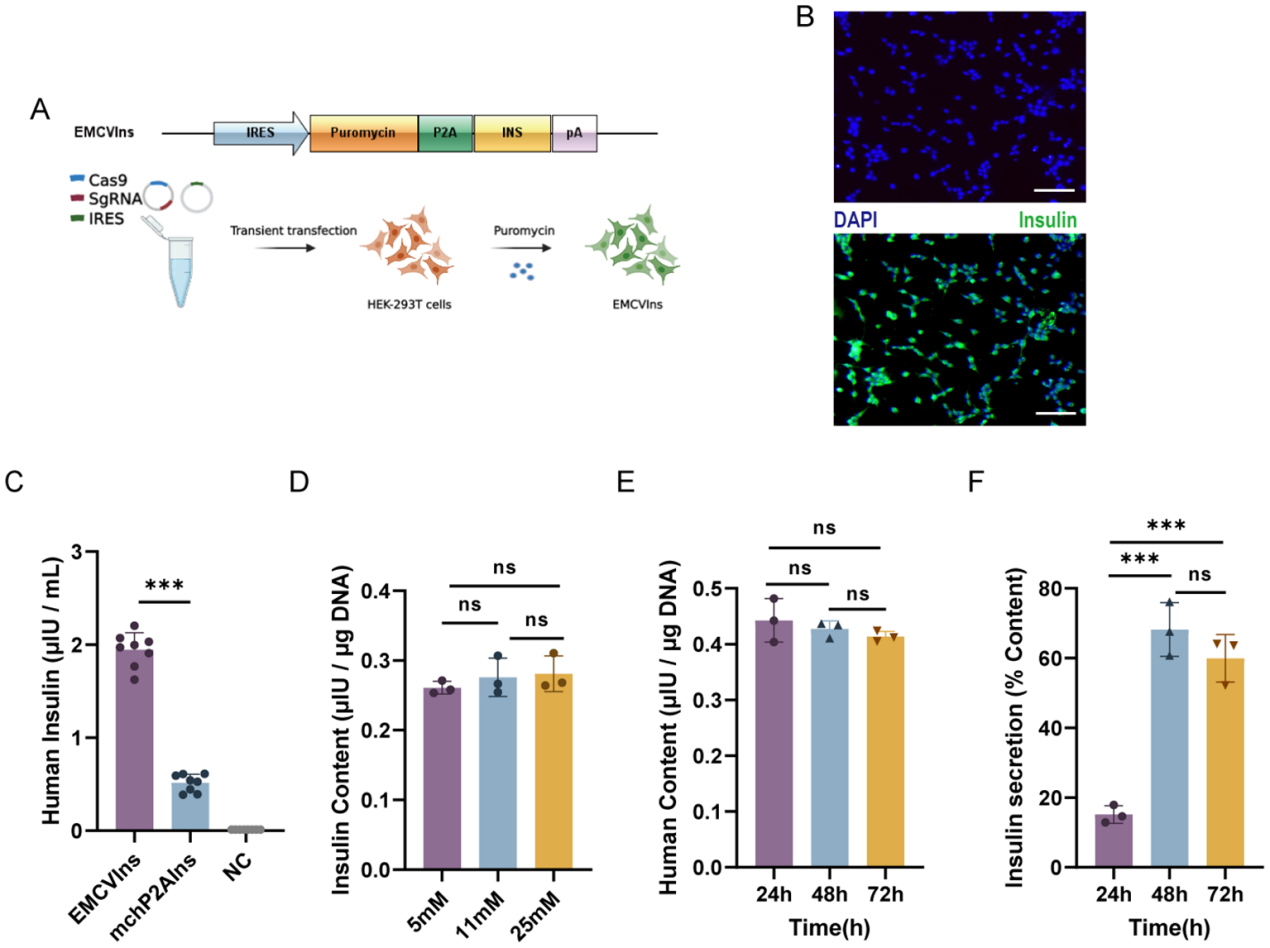
Engineering of stable EMCVIns cells for exogenous insulin. **(A)** Schematic of modified donor plasmids and progression to obtain stable insulin expressing EMCVIns cells. **(B)** Insulin expression was analyzed with immunofluorescence. Green fluorescence indicated human insulin. Scale bar: 50μm **(C)** Supernatant Insulin level produced by EMCVIns cells and mchP2AIns cells were assessed using ELISA. Total insulin content by EMCVIns cells at different concentrations of **(D)** glucose and **(E)** incubation times. **(F)** Insulin secretion from engineered cells at different times. Fresh culture medium was used as negative control (NC). Data are expressed as mean ± SD. n = 5; **p < 0.01, ***p < 0.001 by student’s t-test, one-way ANOVA and Tukey post-test.

To obtain pure lines of engineered insulin-producing cells, we replaced the mCherry sequence in the ires-mchP2AIns (+HAs) donor plasmid with a puromycin DNA sequence, creating the EMCVP2AIns (+HAs) donor plasmid. We then co-transfected Cas-sg1 with the EMCVP2AIns (+HAs) donor plasmid into HEK-293T cells, as previously described. The cells were subsequently screened with 10 µg/mL of puromycin for five passages to obtain pure lines of insulin-secreting HEK-293T cells, designated as EMCVIns (**Figure 2A**). Immunofluorescent staining confirmed insulin expression in EMCVIns cells (**Figure 2B**).

Then, to evaluate whether the CRISPR/Cas9-induced HDR-mediated modified insulin gene integration in EMCVIns cells could successfully secrete mature insulin into the culture medium, both EMCVIns cells and their supernatant were analyzed by ELISA. The mature insulin production detected in the EMCVIns culture medium after stable transfection was 1.95 ± 0.26 μIU·10^5^cells^-1^·mL^-1^·24 h^-1^ (**Figure 2C**). EMCVIns cells were then stimulated with different concentrations of D-glucose (5mM, 11mM, 25mM), and ELISA was used to assess changes in insulin levels. The results showed no significant changes in insulin levels with varying glucose concentrations (**Figure 2D**). Furthermore, we measured the total insulin synthesis versus secretion levels of engineered EMCVIns cells at 24 h, 48 h, and 72 h after cell seeding. The data indicated that the total insulin synthesis level did not increase over time (**Figure 2E**). However, the insulin secretion level increased with time and peaked at 48 hours (**Figure 2F**). These results suggest that mature insulin can be successfully synthesized and secreted into the supernatant using the CRISPR/Cas9-induced HDR-mediated gene integration method.

### Cytopore I is favorable for EMCVIns microencapsulation

**Figure 3:**
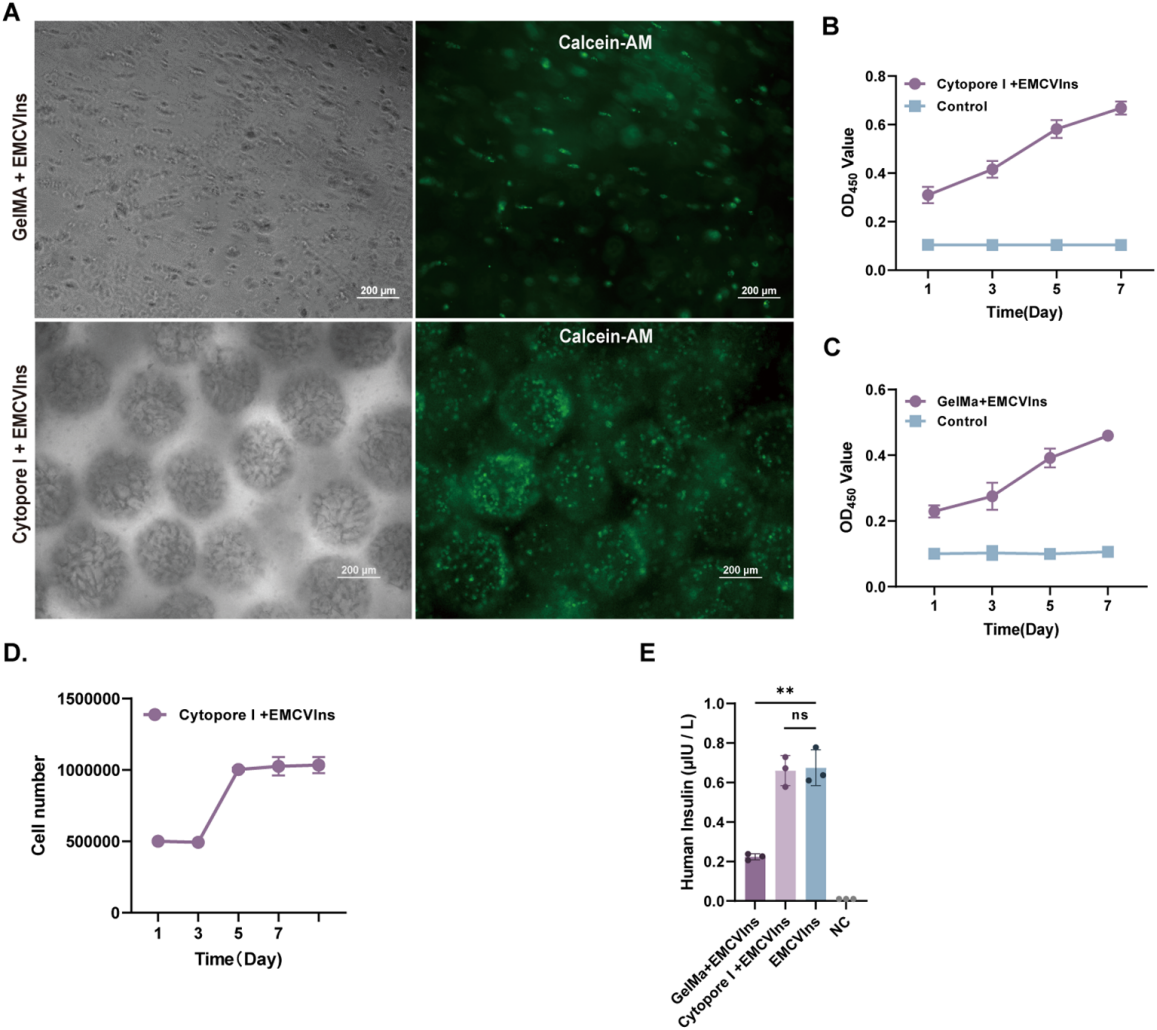
Microencapsulation supports the proliferation of EMCVIns cells. **(A)** Morphology of GelMA and Cytopore I encapsulated EMCVIns cells were imaged under bright field and fluorescence by inverted microscopy. Green fluorescence indicated living cells by Calcein-AM. Scale bar: 200 μm. The relative cell proliferation of **(B)** Cytopore I and **(C)** GelMA encapsulated EMCVIns cells were determined by CCK8 assay, respectively. **(D)** The absolute cell viability of Cytopre I encapsulated EMCVIns cells were tested using Crystal violet staining. Unencapsulated cells cultured in a 2D environment served as control. **(E)** Insulin level in the different microencapsulation group was checked by ELISA. An equal number of EMCVIns cells cultured in normal 2D-culture conditions was used as positive control, while fresh culture medium was used as negative control (NC). Data are expressed as mean ± SD. n = 3; **p < 0.01 by student’s t-test and one-way ANOVA.

The triggered immune response is an unavoidable and crucial factor that must be considered during implantation (46, 47). To minimize immune responses potentially triggered by EMCVIns cell engraftments, the cells were encapsulated using Cytopore I or GelMA-60 at a density of 5 x 10^5^ cells/mL. To assess the survival of EMCVIns cells post-encapsulation, Calcein-AM staining was used to label living cells within the capsules. Both materials were found to carry living cells (indicated by green fluorescence), with Cytopore I microcarriers encapsulating a greater number of living cells (**Figure 3A**). The relative cell proliferation of EMCVIns cells encapsulated in both materials was determined using a CCK-8 assay by measuring absorbance at 450 nm. Cytopore I-encapsulated cells exhibited greater proliferative ability compared to GelMA-60 (**Figures 3B, C**), and viability was also confirmed by crystal violet staining (**Figure 3D**). To ensure continuous insulin secretion and to evaluate whether the encapsulation materials could hinder insulin secretion *in vitro*, the culture supernatant was collected and analyzed by ELISA. As shown in **Figure 3E**, the mature insulin production in the conditioned medium was 0.22 ± 0.02 μIU·10^5^ cells^-1^·mL^-1^24 h^-1^ and 0.66 ± 0.08 μIU·10^5^ cells^-1^·mL^-1^24 h^-1^ for Cytopore I-encapsulated EMCVIns cells. Overall, compared to GelMA-60, Cytopore I was superior for cell survival, proliferation, and did not interfere with insulin secretion, making it the preferred choice for further *in vivo* studies.

### Implantation of insulin-secreting EMCVIns into streptozotocin-induced diabetic mice ameliorated hyperglycemia

**Figure 4:**
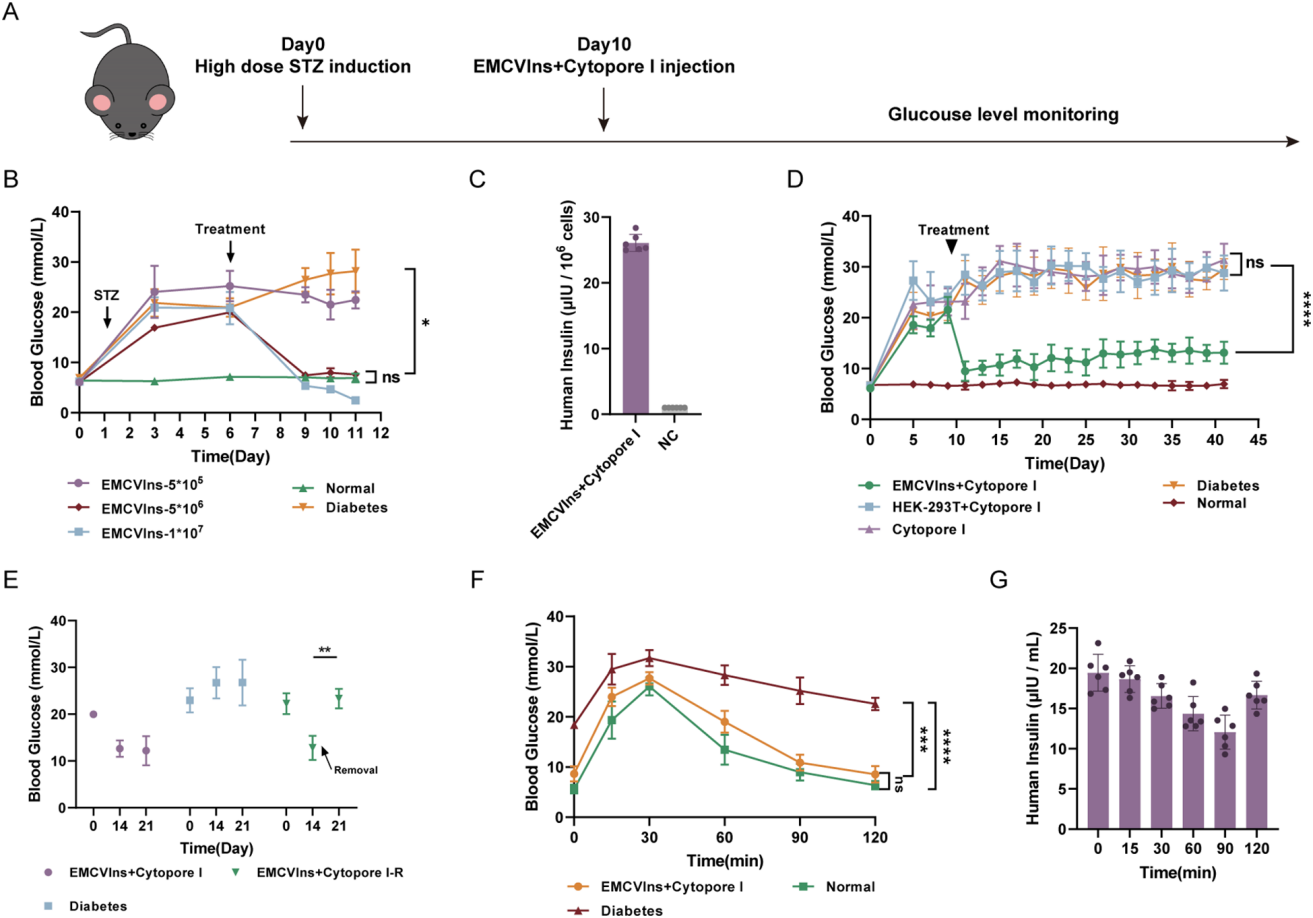
Implantation of Cytopore I encapsulated EMCVIns cells ameliorated hyperglycemia in diabetic mouse models. **(A)** Schematic timeline of diabetic mice model induction and implantation treatment. **(B)** Various numbers of EMCVIns cells were encapsulated into Cytopore I microcarriers and given to STZ-induced diabetic mice. Blood glucose was monitored at indicated time points. **(C)** The quantity of insulin produced by 5x10^6^ EMCVIns cells encapsulated in Cytopore I was determined by ELISA. A fresh culture medium was used as negative control (NC). **(D)** An equal number (5x10^6^) of EMCVIns and HEK-293T cells were encapsulated by Cytopore I and implanted into an inguinal fat pad in STZ-induced diabetic mice, respectively. Blood glucose was monitored at indicated time points. **(E)** In the group of EMCVIns+ Cytopore-R, the implanted EMCVIns cells were removed from treated mice on day 14 as indicated by the arrows. Blood glucose was monitored at indicated time points. Blood **(F)** glucose and **(G)** human insulin levels were monitored at the indicated time point after intraperitoneal glucose stimulation. Data are expressed as mean ± SD.; *p < 0.1, **p < 0.01, ***p < 0.001, ****p < 0.0001 by student’s t-test, one-way ANOVA and Tukey post-test.

Diabetic mice were generated by administering streptozotocin (STZ) at a dose of 40 mg/kg to C57BL/6 mice for five consecutive days, as described in the methods (**Figure 4A**). Hepatic glycogen depletion was confirmed by periodic acid-Schiff reactions in the livers of STZ-treated mice compared to controls (**Supplementary Figure 2B**). Additionally, H&E staining revealed clear pathological and morphological alterations in the pancreas of STZ-treated mice compared to controls (**Supplementary Figure 2A**).

To verify the ability of EMCVIns cells to ameliorate hyperglycemia in the diabetic mouse model, Cytopore I-encapsulated EMCVIns cells were implanted into STZ-induced diabetic mice. Fasting blood glucose levels were significantly reduced after EMCVIns + Cytopore transplantation in a time- and dose-dependent manner (**Figures 4B, D**). Compared to untreated diabetic mice, EMCVIns cells encapsulated in Cytopore I (1×10^7^) effectively reversed blood glucose levels within 72 hours post-implantation. However, this group developed persistent hypoglycemic symptoms and eventually died. In contrast, the group receiving 5×10^5^ EMCVIns cells encapsulated in Cytopore I maintained the current glucose level without further increases. Notably, in the group implanted with 5×10^6^ Cytopore I-encapsulated EMCVIns cells, fasting blood glucose levels remained close to the normal range throughout the experiment without inducing hypoglycemia (**Figure 4B**). Therefore, 5×10^6^ EMCVIns cells were selected for encapsulation and transplantation in further studies. The insulin production by Cytopore+5×10^6 EMCVIns cells reached 26.09 6.0.13 μIU·10^6^cells-1·mL-1 in the culture media, as tested by ELISA (**Figure 4C**).

Remarkably, EMCVIns + Cytopore implantation therapy reversed high blood glucose concentrations in diabetic mice over six weeks, maintaining fasting blood glucose levels at 11.6 ± 2.15 mmol/L (**Figure 4D**). Excision of grafts two weeks after transplantation resulted in a spike in fasting blood glucose levels, reverting to hyperglycemia (**Figure 4E**), confirming the effectiveness of the grafts.

An intraperitoneal glucose tolerance test was conducted on different groups of mice (Cytopore I + EMCVIns-treated STZ-induced mice, STZ-induced diabetic mice, and normal mice) to confirm the ability of EMCVIns cells encapsulated in Cytopore I to maintain blood glucose levels. On day 14, C57BL/6 mice were injected with a 20% glucose solution (2g/kg), and their blood glucose and insulin levels were monitored. Blood glucose levels increased in both the EMCVIns-implanted group and the normal mice group 30 min after glucose stimulation and then recovered, reaching normal levels approximately 2 hours after stimulation (**Figure 4F**). In the diabetic group, blood glucose levels decreased slowly after the initial spike and remained hyperglycemic. Human insulin produced by EMCVIns cells, detected in the serum of the mice, declined within 90 min after glucose stimulation and then gradually rebounded (**Figure 4G**), suggesting its involvement in reducing glucose levels.

**Figure 5:**
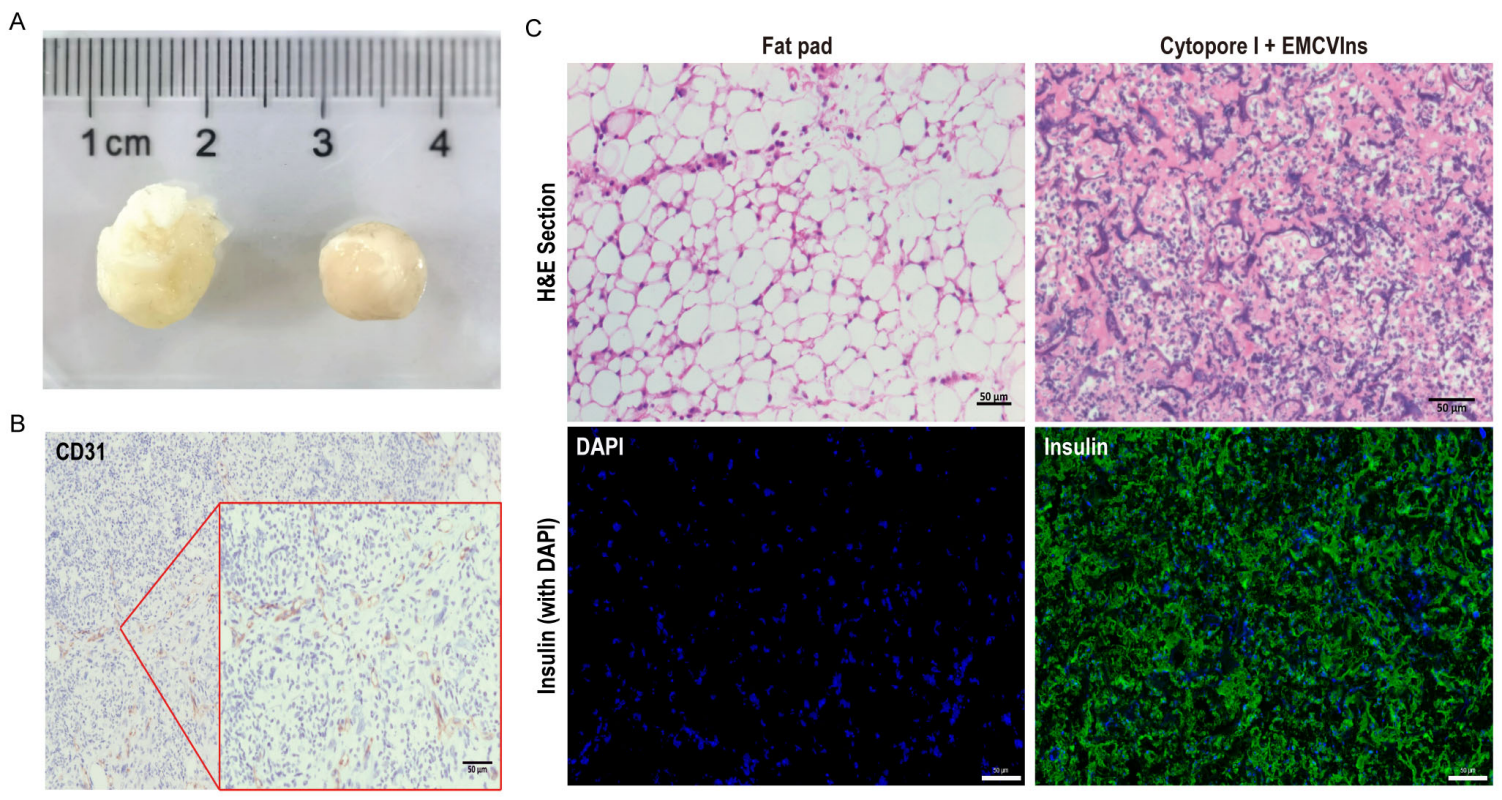
Tissue compatibility of Cytopore I microcarriers. 200 μL of Cytopore I encapsulated EMCVIns cells were injected into the inguinal fat pad of C57BL/6 mice. **(A)** Gross view of Cytopore I microcarriers formed structures 14 days after injection. **(B)** IHC staining to check the expression of CD31(platelet endothelial cell adhesion molecule 1) on sectioned Cytopore I microcarriers formed structures. **(C)** H&E staining on sections of the normal fat pad and Cytopore I+ EMCVIns cells- formed structures. Immunofluorescence staining to confirm human insulin expression (Green) on sections of the normal fat pad and Cytopore I+ EMCVIns cells- formed structures. Scale bar: 50 μm.

The tissue compatibility of microcarriers was examined two weeks post-implantation by retrieving the grafts for further analysis. As shown in **Figure 5A**, the implanted grafts were encapsulated by the host’s adipose tissue, forming a solid, tissue-like structure with its own blood supply. The implants were then paraffin-embedded and sectioned for immunohistochemical (IHC) staining using CD31, a marker for endothelial cells, to detect the presence of endothelial cells in the invaded blood vessels of the excised implants. A strong CD31 signal was observed in the excised implants (**Figure 5B**), indicating successful blood vessel formation within the grafts. H&E staining was performed on the implants to visualize the morphology of encapsulated cells within the tissue-like structure (**Figure 5C**). Additionally, immunofluorescent staining for human insulin in the implant revealed significant insulin expression within the EMCVIns-encapsulated grafts (**Figure 5C**).

**Figure 6:**
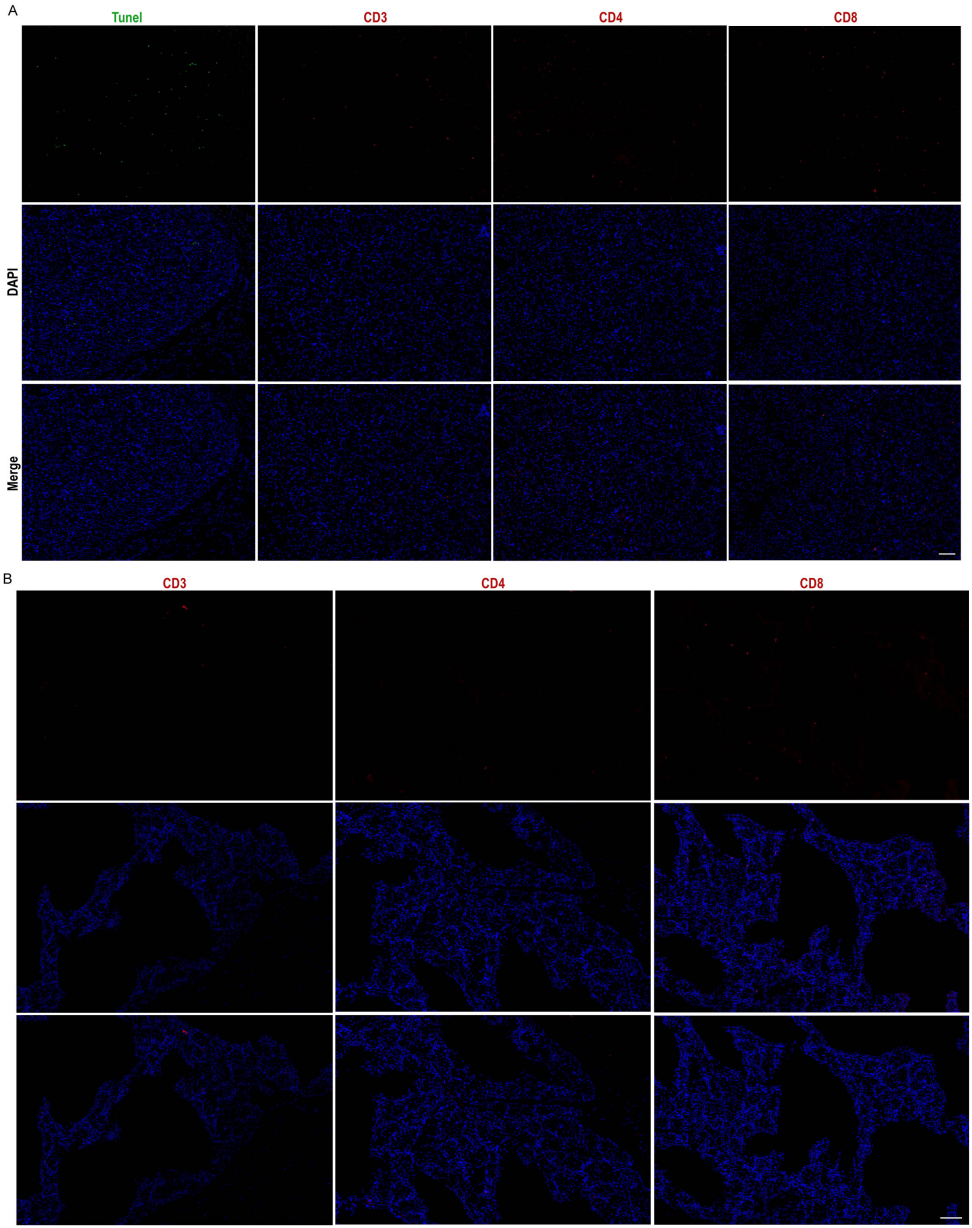
Grafts exhibit good *in vivo* biocompatibility. Cytopore I+ EMCVIns cells Graft **(A)** and normal mouse adipose tissue **(B)** apoptosis factor Tunel (green), immunity factors CD3, CD4, CD8 (red) characterization, and DAPI (blue). Scale bar: 100 μm.

To evaluate the biocompatibility of the grafts, immunofluorescence staining was conducted, including the apoptosis factor TUNEL and immune cell markers CD3, CD4, and CD8, at the conclusion of the experiment. Compared to normal adipose tissue (**Figure 6B**), there was no substantial infiltration of immunological factors in the grafts, indicating that the grafted microspheres provided effective immune isolation. A minimal presence of TUNEL-positive cells suggested a low level of apoptosis within the grafts (**Figure 6A**). In summary, the study results indicate that Cytopore I is biocompatible and exhibits immune isolation effects. The absence of significant immune factor infiltration suggests that this transplantation method has a low risk of inflammation. Moreover, the grafts enable the formation of host blood vessels, which facilitates the exchange of nutrients, including oxygen, between the graft and the host. This environment supports the prolonged survival of engineered cells *in vivo* while preserving the normal insulin secretion function of EMCVIns.

Collectively, our results demonstrate that Cytopore I-encapsulated EMCVIns implants are capable of uninterrupted insulin secretion, which may contribute to the reversal of hyperglycemia and the potential achievement of long-term blood glucose homeostasis.

## Discussion

Despite medical advancements, diabetic patients continue to rely on the invasive infusion of exogenous insulin, primarily insulin analogs, which remain a burden due to the need for multiple dosages. These structurally altered synthetic agonists interact differently with insulin receptors compared to endogenous insulin. Notably, unlike endogenous insulin, synthetic insulin analogs can act at nearly all ligand concentrations under abnormal physiological conditions, leading to shorter or longer receptor stimulation and potentially significant alterations in subsequent signaling and biological effects (48, 49). The use of gene editing to create insulin-secreting engineered cells typically involves introducing strong promoter-mediated insulin-secreting constructs into the genome or cells using lentiviruses or adenoviruses (50–52). However, it has been shown that the intervention of a strong promoter may trigger the silencing or aberrant expression of nearby genes. Additionally, studies have revealed that a vector-borne promoter, intended to drive the expression of the transgene, can be randomly integrated, potentially leading to the unexpected activation of nearby genes, including oncogenes (53, 54).

Site-specific gene integration enables stable expression of exogenous genes, heralding a new era in gene therapy. With the aid of CRISPR/Cas9 technology, targeted DNA breaks can be introduced at specific genomic sites using pre-designed sgRNAs, facilitating precise HDR-based integration that reduces the risk of off-target integration (55, 56). Leveraging this approach, we have constructed a promoter-free, IRES-based expression system that couples insulin expression with the robust expression of GAPDH without disrupting its own expression.

To create a promoter-free endogenous insulin expression system, we synthesized modified insulin genes featuring furin-excisable sites (PC1/3 and PC2 recognition sites modified accordingly) based on previous research (44) These genes were integrated into a specific site of the GAPDH locus in HEK-293T cells using CRISPR-Cas9 tools (**Figures 1A, C, D**). The successful integration and secretion of mature insulin, facilitated by the modified furin-cleavable sites, were confirmed via ELISA (**Figures 1B, E**, **2B**). However, the engineered cells based on this IRES were not glucose-responsive (**Figures 2D, E**). Stimulation with varying glucose concentrations (5mM, 11mM, 25mM) did not result in significant changes in total insulin content, likely because the EMCV-IRES is not inherently sensitive to glucose. To address this, replacing it with a different type of IRES or integrating glucose-sensitive components could be promising, and such work is ongoing. Additionally, the percentage of insulin secretion peaked at 48 hours (**Figure 2F**) and then decreased at 72 hours, possibly due to limited cell proliferation in the culture system.

Microcarriers have been effectively utilized for the culture of anchorage-dependent cells, facilitating easy scale-up and benefiting cell therapy applications (57, 58). Adequate oxygen supply and favorable substance exchange are crucial for cell survival post-transplantation (59). We observed that EMCVIns cells could proliferate and grow on both GelMA and Cytopore I microcarriers (**Figures 3A–D**), with microcarriers being more conducive to cell survival and insulin secretion. Encapsulation in GelMA resulted in insulin secretion levels in the medium supernatant that were less than one-third of those detected under normal conditions, possibly due to the electrostatic interaction between the negatively charged GelMA hydrogel and the positively charged insulin protein (**Figure 3E**).

Cytopore I microcarriers demonstrated an exceptional ability to form tissue-like structures that support encapsulated transplanted cells with an appropriate blood supply (**Figure 5B**). After extended *in vivo* transplantation, the cell-carrying microspheres were securely enveloped by the host’s inguinal fat pad, creating a stable and robust fat inclusion body, free from vacuolar structures caused by apoptosis (**Figures 5A,C**). These inclusions simplified the localization of grafts in mice and could be removed as needed, potentially reducing immune risks associated with transplantation (57, 60). The cells within the grafts exhibited healthy growth, with a substantial amount of insulin detected (**Figure 5C**), and minimal apoptotic factors were observed at the end of the experiment, indicating active cell proliferation (**Figures 6A, B**). Throughout the six-week study, fasting blood glucose levels in the transplanted mice were maintained at 11.6 ± 2.15 mmol/L, representing a significant decrease compared to diabetic mice (25.16 ± 4.8 mmol/L) and reversing hyperglycemia [fasting blood glucose ≥ 16 mmol/L is considered to be diabetic (61)]. Upon graft removal, fasting blood glucose levels in the de-transplanted group rebounded to over 16 mmol/L (**Figure 4E**), strongly illustrating the hypoglycemic effect of the engineered cells.

To simulate the changes in blood glucose profile after feeding in mice, we conducted an IPGTT. The results were encouraging, as the blood glucose fluctuations in the EMCVIns-implanted group were comparable to those in the normal group. Following an intraperitoneal injection of glucose, the blood glucose levels in the mice increased rapidly, peaking at 30 min, and then declined until they stabilized at 2 hours, eventually returning to normal (**Figure 4F**). This indicates that the implanted EMCVIns cells have a beneficial hypoglycemic effect *in vivo*. However, it is disappointing that the current engineered EMCVIns cells did not exhibit glucose-sensing mediated regulation of insulin secretion, mirroring the *in vitro* experimental results. The observed decrease and subsequent increase in human insulin levels in the mice may be attributed to the continuous secretory nature of EMCV-IRES. Initially, insulin is used to equilibrate with the additional high glucose load, leading to depletion and then gradual recovery. Additionally, this may be due to the absence of insulin vesicle structures in the engineered cells, unlike β-cells (**Supplementary Figure 3**), which prevents the cells from releasing large amounts of stored insulin to address spikes in blood glucose. Further in-depth studies are required to address this limitation.

At the conclusion of the experiment, we assessed the grafts for immune factors, including CD3, CD4, and CD8, and detected only a minimal level of positive expression (**Figures 6A, B**). The mice in the transplantation group exhibited no signs of inflammation, such as skin ulceration or swelling, and maintained smooth hair and normal body condition. These findings imply that the transplantation of cell-carrying microspheres into the groin is a relatively safe approach. Both the fat pads and the microspheres may provide a degree of immune isolation for the engineered cells, which is beneficial for their long-term survival and the maintenance of their normal function within the host body.

In conclusion, this study—the first to demonstrate that pre-inoculation of IRES-mediated insulin-secreting cells on microcarriers lowers blood glucose in T1D diabetic mice—presents several significant findings: (i) It introduces a promoter-free protein expression system that does not interfere with the host’s gene expression. (ii) It proposes a convenient and effective method of cell transplantation that has not triggered significant immune rejection, suggesting the potential for long-term *in vivo* functionality. (iii) It establishes a correlation between insulin production and the number of cells, indicating that the degree of blood glucose regulation can be modulated by adjusting the number of transplanted cells. (iv) It shows post-feeding glycemic kinetics comparable to those of a healthy group, suggesting that this approach may offer greater therapeutic potential for diabetes than long-acting or fast-acting insulin.

## Data Availability

The original contributions presented in the study are included in the article/**Supplementary Material**. Further inquiries can be directed to the corresponding authors.
