# Supplementary material for: Engineered IRES-mediated promoter-free insulin-producing cells reverse hyperglycemia
Source: Front Endocrinol (Lausanne). 2024 Aug 30;15:1439351. doi: 10.3389/fendo.2024.1439351 (PMC11392723; doi:10.3389/fendo.2024.1439351)
Supplement: Supplementary file 1 [file DataSheet1.pdf]

Supplementary Tables

Table S1 sgRNA sequences used in the study.

**sgRNA**	**Target Sequences**	**PAM**	**Gene Locus**
Sg-1	GAGAGAGACCCTCACTGCTG	GGG	GAPDH 3'-UTR
Sg-4	GCCATGTAGACCCCTTGAAG	AGG	GAPDH 3'-UTR

Table S2 Primers used in the study.

**Name**	**Sequence** (5'-3')	**Use**
F1	GGAGTCCACTGGCGTCTTCA	To amply genomic DNA fragment
R1	AGACCCTAGAATAAGACAGGA	
F2	GCCTTTGTGAACCAACACCTG	
R2	GAGGAGAACATACCAGGTCCC	
F3	GGAATAAGGCCGGTGTGCGTT	
R3	CAGCTGGTAGAGGGAGCAGAT	
F4	AAGCTGACCCTGAAGTTCATC	
R4	AAGGAAAACCACGTCCCCGTG	

Supplementary Figures

Figure.S1

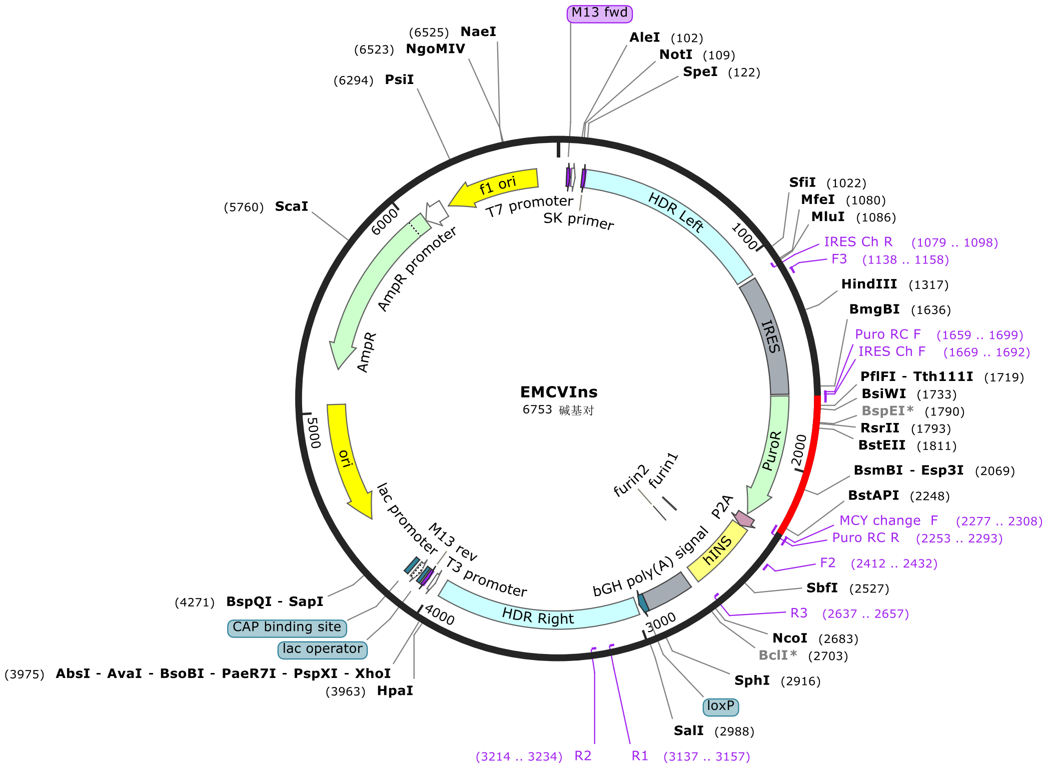

Fig.S1 Schematics of EMCVIns donor plasmid.

Figure.S2

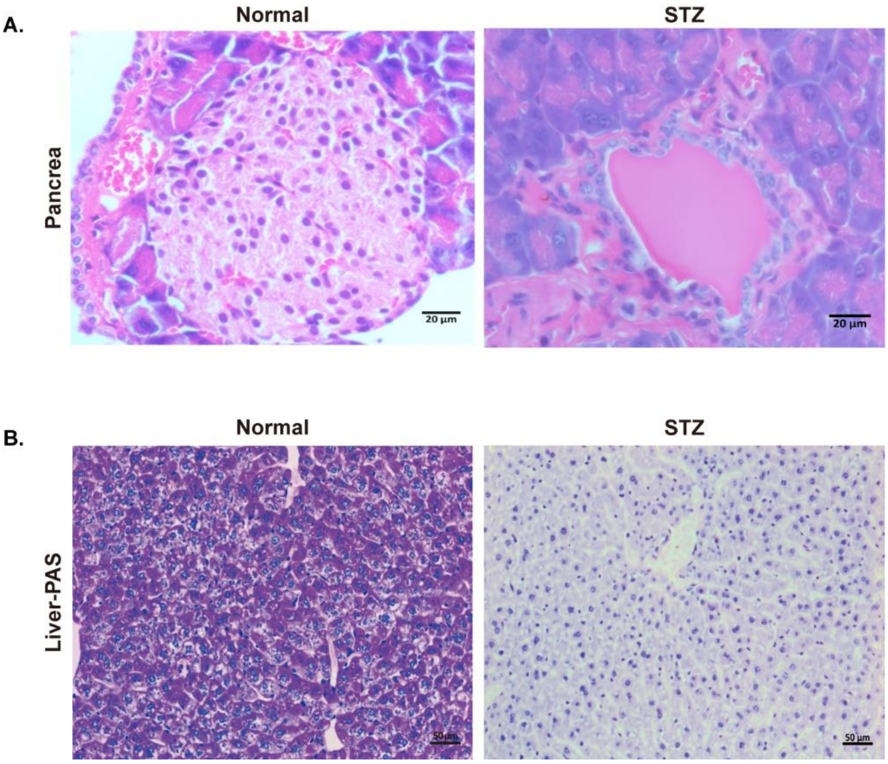

Fig.S2 STZ-induced diabetic mice model. (A)H&E staining on sectioned pancreas from normal and STZ-induced diabetic mice. (B) PAS staining on sectioned liver from normal and STZ-induced diabetic mice.

Figure.S3

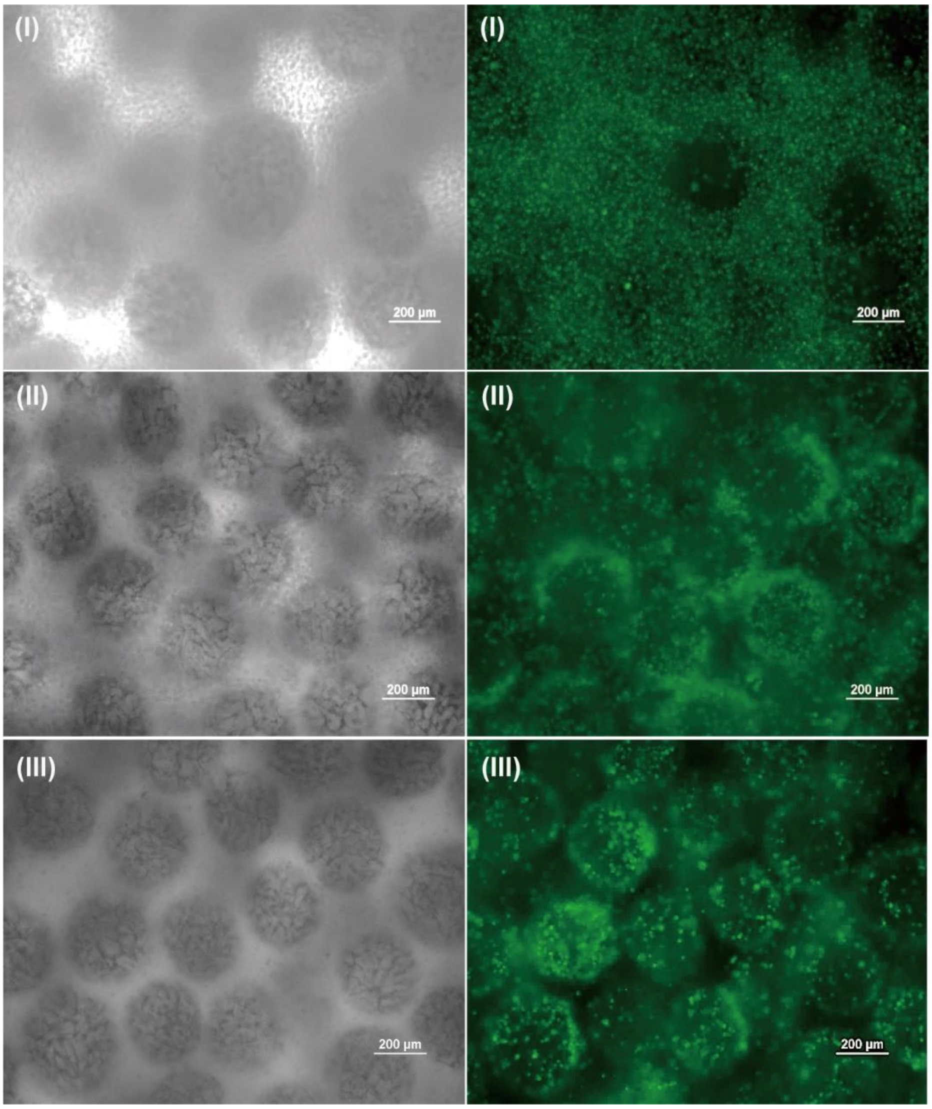

Fig.S3 EMCVIns cells encapsulation by Cytopore I microcarriers. EMCVIns cells were stained with DiO dye (Green), gently mixed with primed Cytopore I microcarriers and seeded on the plates. Along with time, cells gradually entered into the microcarriers. (I-40min, II-80min,III-120min).

Fig.S3

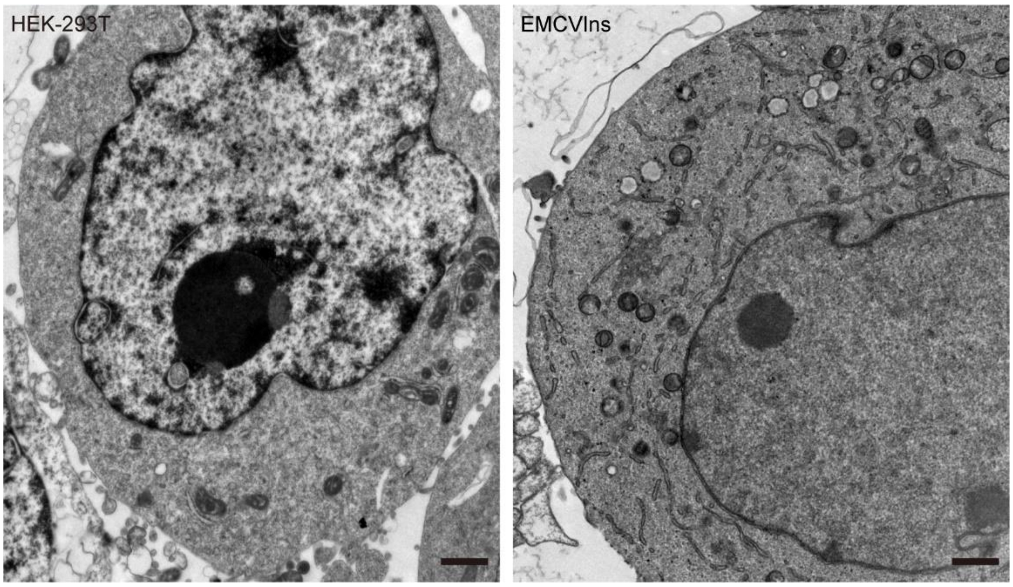

Fig.S3 Intracellular electron micrographs of typical insulin vesicle-like structures were not seen in both HEK-293T and EMCVIns cells, , scale bar:1 μm.
